# Application of Supercooling for the Enhanced Shelf Life of Asparagus (*Asparagus officinalis* L.)

**DOI:** 10.3390/foods10102361

**Published:** 2021-10-04

**Authors:** Youngsang You, Muci Li, Taiyoung Kang, Youngbok Ko, Sangoh Kim, Seung Hyun Lee, Soojin Jun

**Affiliations:** 1 Department of Human Nutrition, Food and Animal Sciences, University of Hawaii at Manoa, Honolulu, HI 96822, USA; youngsang1015@gmail.com (Y.Y.); limuci@hawaii.edu (M.L.); taiyoung@hawaii.edu (T.K.); koyoung27@gmail.com (Y.K.); 2 Department of Food Engineering, Dankook University, Cheonan 31116, Korea; 3 Department of Plant and Food Engineering, Sangmyung University, Cheonan 31066, Korea; samkim@smu.ac.kr; 4 Department of Biosystems Machinery Engineering, Chungnam National University, Daejeon 34134, Korea; seunglee2@cnu.ac.kr

**Keywords:** asparagus, supercooling, pulsed electric field, oscillating magnet field, preservation

## Abstract

Freezing extends the shelf-life of food by slowing down the physical and biochemical reactions; however, ice crystal formation can result in irreversible damage to the cell’s structure and texture. Supercooling technology has the potential to preserve the original freshness of food without freezing damage. In this study, fresh asparagus was preserved in a supercooled state and its quality changes such as color, weight loss, texture, chlorophyll and anthocyanin content, and enzymatic activities (superoxide dismutase and catalase) were evaluated. The asparagus samples were successfully supercooled at −3 °C with the combination treatment of pulsed electric field (PEF) and oscillating magnetic field (OMF), and the supercooled state was maintained for up to 14 days. Asparagus spears preserved in the supercooled state exhibited lower weight loss and higher levels of quality factors in comparison to the frozen and refrigerated control samples.

## 1. Introduction

Asparagus (*Asparagus officinalis* L.) is one of the most widely consumed vegetables in the US due to its unique flavor, low calories, and high nutritional values. Moreover, anthocyanins are one of the largest and most important groups of water-soluble pigments in most species in the plant kingdom. They are accumulated in cell vacuoles and are primarily responsible for diverse pigmentation from orange to red, purple, and blue. Anthocyanins are one of the important groups of phenolic compounds, presenting in fruits and vegetables. They contribute to the characteristic color and have been linked to antihyperglycemic, anticancer, and antimutagenic health benefits [1,2,3,4]. Asparagus has a limited shelf-life of fewer than 5 days at ambient temperatures, mainly due to its high respiratory rate after harvesting [5]. During storage, asparagus undergoes undesirable physiological and compositional changes such as moisture loss, chlorophyll degradation, and lignification [5,6] that lead to a deterioration of the overall quality of the vegetable. Emerging technologies such as edible coatings [3,7] and modified atmosphere packaging [4,8] have shown potential in maintaining the quality of asparagus. By utilizing the pretreatment technology, quality deterioration and microbial contamination can be minimized; however, the proper regulation of temperature and humidity needs to be ensured in order to further increase the microbial safety and enhancing the shelf-life of asparagus [9,10].

Storage temperature is the most significant factor affecting the rate of freshness and loss of produce. Based on the biological and chemical reaction kinetic approach, a higher storage temperature will lead to a quicker loss of quality [11]. For instance, fresh asparagus stored at lower storage temperatures (0 °C and 2 °C) exhibited higher ascorbic acid concentrations than those stored at a higher temperature (4 °C and 6 °C) [12]. Furthermore, it is required that harvested asparagus should be cooled as quickly as possible to reduce the post-harvest heat accumulation, which potentially shortens the shelf-life of the vegetable [11]. In general, refrigeration below 4 °C is recommended for cold storage of fresh vegetables, while freezing is not because ice crystal formation can cause the depolymerization of the cell wall, rupture of the cell membrane, and change in the osmotic pressure, which results in excessive deterioration in the texture after thawing [13].

Supercooling refers to the process of maintaining food unfrozen below its freezing point without ice crystallization. Supercooling has been employed to reduce microbial growth and deteriorative chemical reactions in food by lowering storage temperatures without the loss of structural integrity [14]. James et al. (2009) have demonstrated that unpeeled garlic bulbs could be supercooled at around −6 °C for 1 week, and the supercooled garlic showed no differences in visual appearance between refrigerated samples (1 °C) while freezing damage was found in the frozen garlic bulbs (−30 °C) [15]. However, maintaining a stable supercooled state within foods for an extended period may be particularly problematic due to the random nature of ice nucleation [14]. Recently, electric and magnetic fields have been utilized to control ice nucleation during the freezing process [16,17]. It was found that external electric and magnetic fields may affect the onset of ice crystal formation during freezing and supercooling processes because water consists of dipole molecules and is also diamagnetic. Therefore, water molecules that naturally present in food tend to realign and re-orientate under electric and magnetic fields, meaning that they are potentially able to prevent the ice crystallization process and may lead to a substantial change in the supercooling behavior of food products [18,19,20]. A variety of food products such as beef, chicken breast, fish, and pineapple were supercooled for the extended shelf life with maintained freshness [21,22,23,24]. However, there have been no reports on supercooling for green loose-leaf vegetables using electric and magnetic fields in food preservation.

In this study, the supercooling technique, combining pulsed electric fields (PEF) and oscillating magnetic fields (OMF), was applied to test asparagus for quality factor analysis and quality parameters of preserved asparagus were measured and compared with the control samples stored under refrigeration and freezing. The quality parameters measured to determine the asparagus quality included color, texture, drip loss, and diverse chemical contents.

## 2. Materials and Methods

### 2.1. Sample Preparation

Fresh green asparagus spears were purchased at local grocery stores. Fresh asparagus was cut into a length of 12.5 cm to fit the supercooling chamber. A similar sample weight (50 g) was used in each experimental trial. Control samples in an approximately equal shape and weight were placed at the dummy chambers (without OMF electromagnets and PEF electrodes). All of the asparagus samples were covered with polyethylene (PE) film to avoid dehydration during experiments. A total of three experiments were conducted with the asparagus samples at different storage conditions: (1) refrigeration (4 °C), (2) freezing (−18 °C), and (3) supercooling (−3 °C). Samples were stored for 7 and 14 days. The combined PEF and OMF supercooling treatment, along with control preservations, were performed in triplicate.

### 2.2. PEF and OMF Treatment for Supercooling

**Figure 1:**
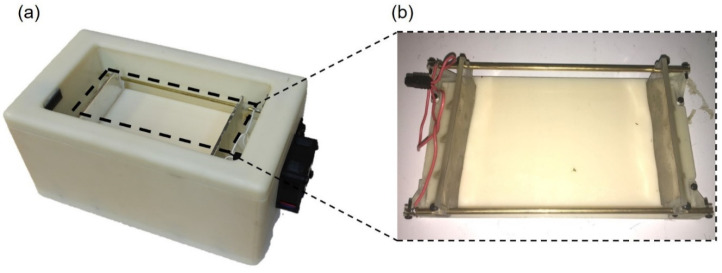
Photos of supercooling device. (**a**) Top view of the supercooling chamber. (**b**) The voltage was applied between the titanium electrodes for PEF. A further detailed device setup can be found in our previous work [21,22].

**Figure 2:**
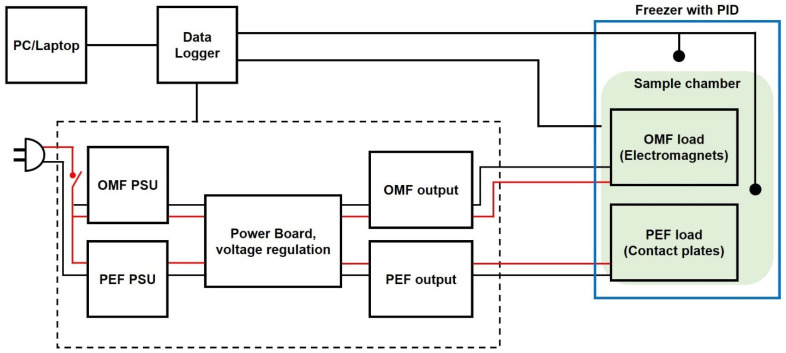
A simplified block diagram of the control unit’s overall system design. PC/Laptop are interfaced with control unit. The external chamber houses PEF electrodes and electromagnets.

In order to maintain the supercooled state for an extended shelf-life period, a custom-designed module to supply both PEF and OMF was fabricated (Figure 1). A detailed device setup can also be found in our previous work [21,22]. The PEF was delivered to the samples via side electrodes made with 0.01 mm thick 99.6% titanium foil (ESPI Metals, Ashland, OR, USA) attached to the sidewalls of the acrylic sample holder. The entire device was placed in a chest freezer (Haier, Qingdao, China). The temperature of the freezer was controlled using a digital temperature controller. All of the temperatures of the samples were collected using a data acquisition unit (DAQ, Agilent 39704A, Agilent Technologies, Inc., Santa Clara, CA, USA). The temperatures of the samples were collected in real-time using T-type thermocouple wires. The degree of supercooling to which the test sample was exposed during treatment was controlled using the identical PEF sequence of the duty cycle of 0.5. The input voltage and frequency of PEF were 20 V and 20 kHz, respectively, in order to keep the internal temperature of the asparagus samples at approximately −4 °C throughout the entire storage period. The applied voltage and frequency of the OMF were 60 V and 1 Hz, respectively. A simplified block diagram of the whole control unit design is shown in Figure 2.

### 2.3. Weight Loss

The weight loss of asparagus samples after storage was determined with a commonly used method [3]. After cutting, fresh asparagus was weighed and recorded as the initial weight. After each preservation treatment, the samples were weighed again and recorded as the final weight. For the frozen samples, asparagus was thawed in the refrigerator (4 °C) for 24 h before their final weights were recorded. Weight loss was calculated as the following equation:

(1) Weight loss (%)=[(Initial weight−Final weight)/Initial weight]×100

### 2.4. Color Analysis

A computer vision system was used to measure the color of asparagus samples [25]. In brief, images were obtained using a digital camera (Canon EOS Rebel T3i, Canon USA Inc., Melville, NY, USA), which was vertically located at a distance of 20 cm from the samples. A circular light bulb (FC12T9, Phillips, Amsterdam, The Netherlands) was used to achieve a uniform light intensity over the samples in the system. The taken images were analyzed using a MATLAB code (MathWorks, Natick, MA, USA) to convert the digital RGB data into the L*a*b* color components.

For sample comparison, the net color difference was calculated using the given codes. The color difference (Δ*E*) was calculated using the equation:

(2) $$\Delta E=\sqrt{{\left(L_{1}-L_{2}\right)}^{2}+{\left(a_{1}-a_{2}\right)}^{2}+{\left(b_{1}-b_{2}\right)}^{2}}$$

Subscripts 1 and 2 indicate the color components before and after the treatment, respectively. Measurements were taken at ten different locations on the sample and the average was calculated.

### 2.5. Texture

The texture measurement was performed by following the method of Kidmose & Kaack with slight modifications [26]. All of the asparagus spears were kept at room temperature before the test. Shear force values were measured using a TA-XT2 texture analyzer equipped with a Warner-Brazler shear force cell with a single blade and a blade speed of 10 cm/min [27]. A total of 3 spears were measured for each of the two replications, and each spear was measured 3 times with a 2 cm interval from the butt end. The peak cutting force was recorded at each 2 cm interval, and the average peak cutting force was calculated. The shear force values (g/m), which are an expression of toughness, were calculated as the average peak cutting force: spear divided by the spear butt.

### 2.6. Chlorophyll

The measuring method of the chlorophyll content in the asparagus was utilized, with slight modification of Wu & Wang’s method [28]. In brief, 2 g asparagus was ground in a mortar and extracted in 10 mL of 95% (*v*/*v*) ethanol and centrifuged at 6000 rpm for 15 min. Every step was performed at 4 °C. The supernatant was used to determine the chlorophyll content. Chlorophyll quantification was performed spectrophotometrically using a spectrophotometer at 665 and 649 nm, and the chlorophyll content was expressed as chlorophyll mass on a fresh weight (FW) basis (mg/kg FW). The calculation of chlorophyll amount was described by Sumanta et al. (2014) as follows [29]:

(3) $${\mathrm{Ch}}_{a}=13.36\times A_{664}-5.19\times A_{649}$$

(4) $${\mathrm{Ch}}_{b}=27.43\times A_{649}-8.12\times A_{664}$$

(5) $$\mathrm{Chl}={\mathrm{Ch}}_{a}+{\mathrm{Ch}}_{b}$$

A = Absorbance, Ch*_a_* = Chlorophyll a, Ch*_b_* = Chlorophyll b.

### 2.7. Anthocyanin

The anthocyanin content was determined on the asparagus as described by Tzoumaki et al. (2009), with some modifications [3], which were initially proposed by Flores et al. (2005) [30]. Firstly, 2 g of asparagus sample tissue was chopped in a mortar and extracted with 5 mL of extractant solution which was composed with 99.98% ethanol/0.5 N HCl (85:15 *v*/*v*). Then, the homogenate was incubated for 4 h in darkness. Every step was carried out at a temperature of 4 °C. The homogenate was then centrifuged at 6000 rpm for 30 min, and the supernatant was used to perform the spectrophotometric measurement at 533 nm using the extraction medium as the blank. The results were expressed as mg anthocyanins per g FW of asparagus.

### 2.8. Superoxide Dismutase (SOD) Activity

SOD in asparagus samples was extracted via the method employed by An et al. (2007) [31]. With 10 mL extraction buffer, 2 g of asparagus samples were ground in a mortar. The content of extraction buffer was composed with 50 mM phosphate buffer, pH 7.8 containing 0.1 mM EDTA, 0.3% (*w*/*v*) TrintonX-100, and 4% (*w*/*v*) polyvinylpolypyrrolidone (PVPP). The mixture was centrifuged at 6000 rpm for 30 min and the supernatant was used as the crude enzyme extract. Every step was performed at a temperature of 4 °C. Then SOD activity was measured using a slightly modified previous method [32,33]. SOD activity was measured by measuring its ability to inhibit the nitro blue tetrazolium (NBT) photochemical reduction. A sum of 50 microliters of enzyme extract were added into 3 mL of reaction buffer, which contained 50 mM phosphate buffer (pH 7.8), 13 mM methionine, 75 µM NBT, 2 µM riboflavin, and 0.1 mM EDTA. Then, the mixture was placed below a fluorescent lamp for 20 min. The absorbance of the reaction mixture was measured at a wavelength of 560 nm. Furthermore, 1 unit of SOD was considered as the amount of enzyme that inhibited NBT reduction by 50%.

### 2.9. Catalase (CAT) Activity

CAT activities in asparagus samples were measured using a slightly modified method from W. X. Li et al. (2008) [33]. A total of 2 g of asparagus samples were ground in a mortar in 10 mL extraction buffer and then centrifuged at 6000 rpm for 15 min at 4 °C. The CAT extraction buffer contained 0.2 M phosphate buffer, pH 7.8 containing 1% (*w*/*v*) PVPP. After centrifugation, the supernatant was used as CAT activity measurement. The CAT activity was analyzed using the method of Aebi (1984), with some modifications [34]. A sum of 200 microliters of enzyme extract were added to the reaction mixture, which contained 1.5 mL of phosphate buffer (0.2 M, pH 7.8), 1 mL of distilled water, and 0.3 mL of H_2_O_2_ (0.1 M). The CAT activity was determined by measuring the rate of disappearance of hydrogen peroxide. The decrease in hydrogen peroxide was followed by a decline in absorbance at 240 nm. One unit of CAT activity was defined as the amount of enzyme, which caused the absorbance decrease of 0.1 at 240 nm/min at room temperature.

### 2.10. Statistical Analysis

All of the results are means ± standard deviation, and the data were statistically evaluated by ANOVA with mean differentiation by Duncan’s multiple range test (α = 0.05). The statistical software was the SPSS (v. 16.0, IBM, Chicago, IL, USA).

## 3. Results and Discussion

### 3.1. Combination PEF and OMF Supercooling Treatments

**Figure 3:**
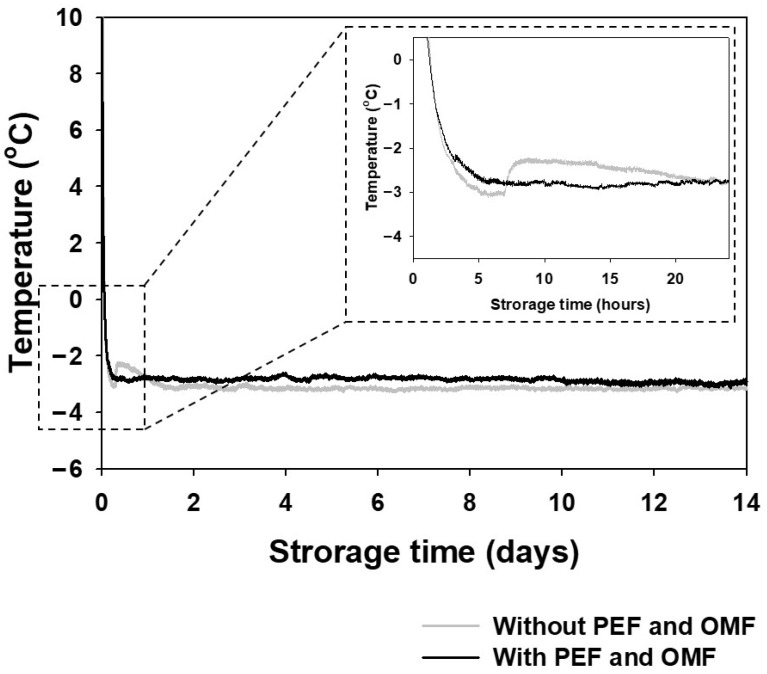
Temperature profiles of supercooled sample (black) and frozen sample (gray) during 14-day storage period.

The cooling curves of the frozen and supercooled asparagus samples are shown in Figure 3. The equilibrium freezing temperature of the asparagus was found at around −2.3 °C. Supercooling is the phenomenon where the temperature of asparagus is decreased below its equilibrium freezing temperature without forming ice crystallization because of an energy barrier. Before the nucleation begins, interfacial tension has to surmount through the external PEF and OMF [35]. Each negative control sample was frozen below freezing point, while the supercooled samples had reached a temperature of −3 °C without ice nucleation and maintained supercooling stage under the combined PEF and OMF during two-week storage.

### 3.2. Color

**Figure 4:**
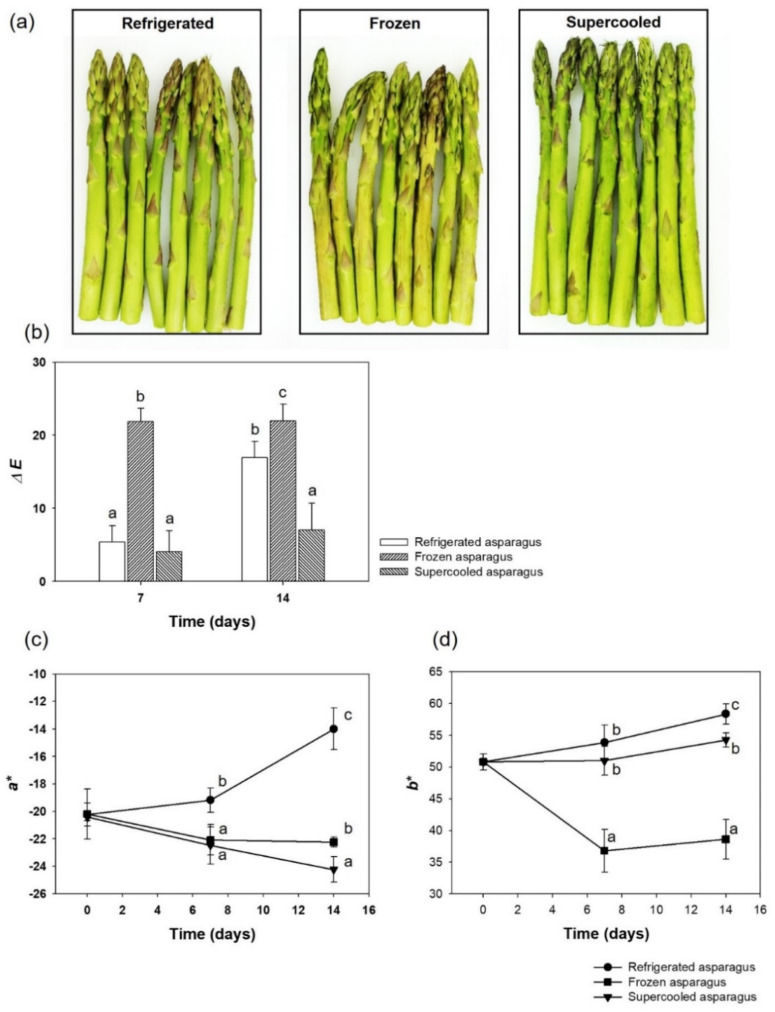
(**a**) Color differences between fresh, refrigerated, and supercooled samples after two weeks of storage. Photos were taken when the samples’ temperature reached room temperature. Mean values of (**b**) ∆*E*, (**c**) a*, and (**d**) b* of samples at 7 and 14 days of storage period under treated conditions. Error bars represent standard deviation and different superscript lowercase letters indicate significant differences (*p* < 0.05) with the same column.

Figure 4a shows the colors of refrigerated, frozen, and supercooled asparagus after two-week storage. The results of color analysis of asparagus samples during preservation were shown in Figure 4b,c with Δ*E*, a*, and b* values. For frozen samples, Δ*E* exhibited a significant change in the first week but showed no significant change thereafter. The color-related ingredients, such as chlorophyll, suffered permanent damage during storage because the extreme temperature fluctuations from freezing (−20 °C) and thawing (4 °C) in frozen asparagus were processed. As for refrigeration samples, the sample showed a steady worsening condition during preservation. The results showed that supercooling had an enhanced effect on color preservation. The delta *E* of supercooling asparagus within 2 weeks was maintained below 5, but the refrigeration value increased up to 15. Moreover, supercooled asparagus can retain a higher greenish color value than refrigerated and frozen samples (Figure 4c), while frozen asparagus sample has the highest yellowish value during storage (Figure 4d). This changing trend of asparagus was closely related to the decrease in chlorophyll and anthocyanin chemical reactions. Compared with other samples, supercooling preservation had some positive effects on preventing color changing.

### 3.3. Drip Loss and Texture

**Figure 5:**
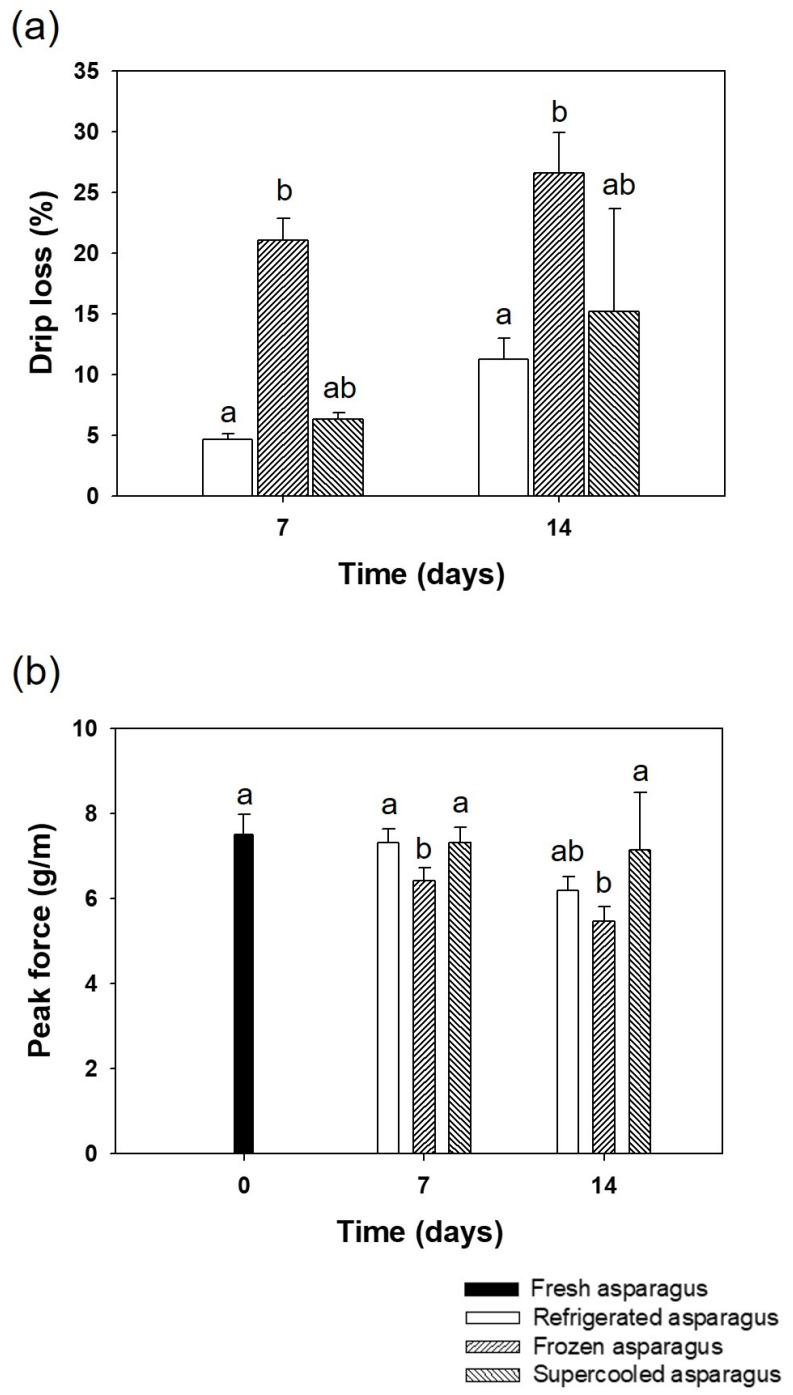
Mean values of (**a**) drip loss and (**b**) texture changes in asparagus samples at 7 and 14 days of storage period under different treatment conditions. Error bars represent standard deviation and different letters indicate significant differences (*p* < 0.05) between treatments at specific time points.

Figure 5a shows the drip loss from the asparagus over the two-week preservation period. The drip loss from the frozen samples was significantly higher than the refrigerated and supercooled samples at each time point of storage. The more significant amount of drip loss in the frozen samples was a direct result of ice damage to the cellular structure of the food samples. In one-week storage, the drip loss results show slight differences between the refrigerated and the supercooled samples.

Figure 5b represents the texture of the asparagus sample stored under different conditions over the two-week preservation period and measured as peak cutting force. In the first week, there was no significant difference among the refrigerated and the supercooled asparagus samples. With the frozen samples, the asparagus had a much softer texture after the freezing and thawing process at −18 °C and 4 °C, respectively.

Texture is one of the common parameters for asparagus quality analysis. Fresh asparagus has a pleasant crispy texture. The texture of asparagus is highly related to fibrousness and the process of hardening that occurs after harvesting; the latter is accompanied by the lignification of the fibers [36]. Besides, changes in texture may also reflect losses in tissue water and increases in other phenolic compounds, apart from lignin. In asparagus spears, the unaltered shoot differentiation also includes thickening and lignification of cell walls in the sclerenchyma ring and in vascular bundles. These processes rapidly result in the undesired toughening of spears [37]. In addition, spear stiffness declined during the entire storage period, i.e., spears became more elastic irrespective of the treatments. Since the fibrousness of asparagus is a key factor determining edible quality, any change in the fiber content due to storage conditions would impact the freshness of asparagus [38]. The large weight loss from spears stored for 14 days is observed due to moisture loss and loss in reducing substances. Maintaining higher humidity in the store can minimize the weight loss to some extent but would be impractical for long-term storage before exporting or retail display.

### 3.4. Chlorophyll

**Figure 6:**
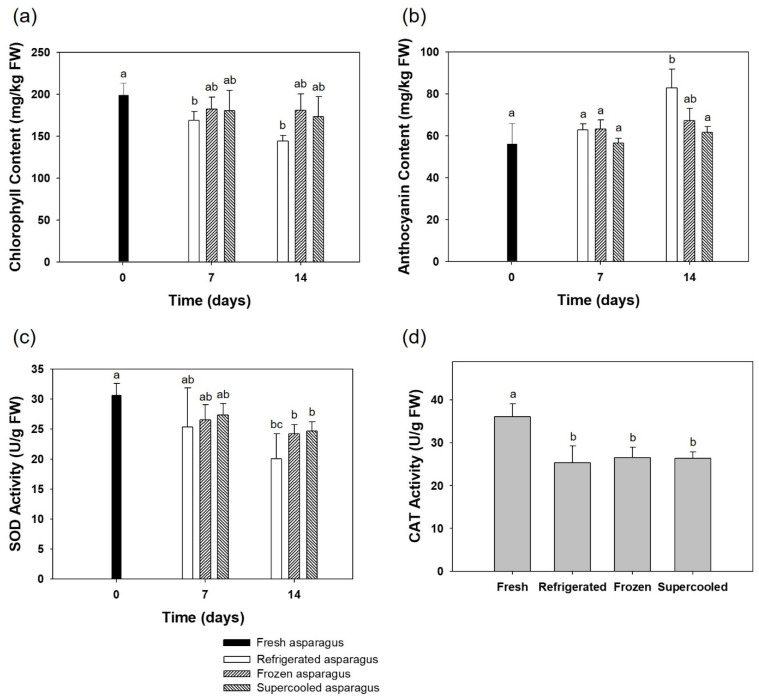
Mean values of (**a**) chlorophyll contents, (**b**) anthocyanin contents, (**c**) SOD activities, and (**d**) CAT activities in asparagus samples over the two-week preservation period. Error bars represent standard deviation and different letters indicate significant differences (*p* < 0.05) within the same storage value.

Figure 6a shows the chlorophyll content of the asparagus over the two-week preservation period. In this study, a significant loss in the chlorophyll content was observed during the storage period in all of the asparagus samples. As a result, supercooling and freezing storage had an outstanding result in maintaining chlorophyll contents in asparagus samples. However, refrigeration storage appeared to be less likely to maintain the chlorophyll content. Supercooling preservation retarded the loss in chlorophyll content and slowed down the oxidative reaction that is responsible for the breakdown of the pigment. According to Sumanta et al. (2014) [29], chlorophyll includes Chlorophyll a and Chlorophyll b. Chlorophyll b differs from Chlorophyll a only in one functional group bonded to the porphyrin ring; moreover, it is more soluble than Chlorophyll a in polar solvents because of its carbonyl group. It was reported that changes in the color of asparagus during the cold storage period were consistent with the contents of Chlorophyll a.

### 3.5. Anthocyanin

Figure 6b shows the anthocyanin content of the asparagus over the two-week preservation period. In this study, a significant increase in anthocyanin was found during the preservation of storage for two weeks. With refrigeration, the amount of anthocyanin was higher than other storage methods, freezing and supercooling, after 14 days of storage. Any significant difference between fresh and supercooled samples during 14 days of storage was not observed.

### 3.6. SOD and CAT Activities

Figure 6c,d show the result of SOD and CAT activities change in asparagus samples in two-week storage. Trends in all of the SOD and CAT activities were decreased during all storage methods. After 14 days of storage, SOD activities of supercooled and frozen samples were significantly higher than refrigerated samples. As for catalase activity, no significant difference was observed. Since it was observed that supercooling samples had a higher SOD activity after 2-week preservation, the supercooling technique process could significantly increase the activities of SOD, lower the accumulation rate of malondialdehyde (MDA) and inhibit the increase in the relative conductivity. In general, free radical production and elimination are always in a dynamic equilibrium state and the free radical level is too low to injure organism call. However, when asparagus undergo a senescence process, the balance of production and elimination could be broken. In a study carried out by W. X. Li et al. (2008) [33], it was found that that SOD and CAT activities of asparagus increased in the beginning and then declined with the extension of storage time under all conditions. Under the hypobaric condition, two indices reached the highest value on the 20th day and then began to slowly decrease [33]. In this study, the SOD and CAT activity was reduced from the first week, which might be because the samples were already displayed at the supermarket for the time being before being purchased.

## 4. Conclusions

A supercooling technique combined with PEF and OMF was applied on subzero temperature storage of asparagus (*Asparagus officinalis* L.) for quality factor analysis. The asparagus was selected because it is popular and considered highly perishable. Asparagus has one-week shelf life when stored under refrigeration, in general. The developed technology was able to extend the shelf life of asparagus for up to 2 weeks whilst fully maintaining the color and texture of fresh asparagus. In addition, supercooled samples showed lower chlorophyll and minimum changes in anthocyanin concentrations, compared with the control refrigeration groups. Supercooling preservation could also significantly increase the activities of superoxide dismutase and decrease the accumulation rate of malondialdehyde. There was a relatively higher drip loss in supercooled asparagus than refrigerated samples; however, the difference was not statistically significant at all. Therefore, the supercooling technology implemented with PEF and OMF functions assured key quality parameters in fresh asparagus while extending its shelf life. It is expected that this technology could offer a radically new food preservation method for consumers and the commercial food vegetable industry.

## Data Availability

Not applicable.
